# Angiotensin II potentiates zinc-induced cortical neuronal death by acting on angiotensin II type 2 receptor

**DOI:** 10.1186/1756-6606-6-50

**Published:** 2013-12-01

**Authors:** Mi-Ha Park, Ha Na Kim, Joon Seo Lim, Jae-Sung Ahn, Jae-Young Koh

**Affiliations:** 1 Neural Injury Research Lab, University of Ulsan College of Medicine, Seoul 138-736, South Korea; 2 Department of Neurology, University of Ulsan College of Medicine, 388-1 Poongnap-Dong, Songpa-Gu, Seoul 138-736, South Korea; 3 Graduate School of Medical Science and Engineering, KAIST, Daejeon 305-701, South Korea

**Keywords:** Zinc, Angiotensin, Neuronal cell death, NAPDH oxidase

## Abstract

**Background:**

The angiotensin system has several non-vascular functions in the central nervous system. For instance, inhibition of the brain angiotensin system results in a reduction in neuronal death following acute brain injury such as ischemia and intracerebral hemorrhage, even under conditions of constant blood pressure. Since endogenous zinc has been implicated as a key mediator of ischemic neuronal death, we investigated the possibility that the angiotensin system affects the outcome of zinc-triggered neuronal death in cortical cell cultures.

**Results:**

Exposure of cortical cultures containing neurons and astrocytes to 300 μM zinc for 15 min induced submaximal death in both types of cells. Interestingly, addition of angiotensin II significantly enhanced the zinc-triggered neuronal death, while leaving astrocytic cell death relatively unchanged. Both type 1 and 2 angiotensin II receptors (AT1R and AT2R, respectively) were expressed in neurons as well as astrocytes. Zinc neurotoxicity was substantially attenuated by PD123319, a specific inhibitor of AT2R, and augmented by CGP42112, a selective activator of AT2R, indicating a critical role for this receptor subtype in the augmentation of neuronal cell death.

Because zinc toxicity occurs largely through oxidative stress, the levels of superoxides in zinc-treated neurons were assessed by DCF fluorescence microscopy. Combined treatment with zinc and angiotensin II substantially increased the levels of superoxides in neurons compared to those induced by zinc alone. This increase in oxidative stress by angiotensin II was completely blocked by the addition of PD123319. Finally, since zinc-induced oxidative stress may be caused by induction and/or activation of NADPH oxidase, the activation status of Rac and the level of the NADPH oxidase subunit p67^phox^ were measured. Angiotensin II markedly increased Rac activity and the levels of p67^phox^ in zinc-treated neurons and astrocytes in a PD123319-dependent manner.

**Conclusion:**

The present study shows that the angiotensin system, especially that involving AT2R, may have an oxidative injury-potentiating effect via augmentation of the activity of NADPH oxidase. Hence, blockade of angiotensin signaling cascades in the brain may prove useful in protecting against the oxidative neuronal death that is likely to occur in acute brain injury.

## Background

Mechanisms of neuronal death associated with acute brain injuries such as ischemia and trauma have been extensively investigated over recent decades [1-3]. Initially, calcium overload induced by glutamate was considered a common mechanism for neuronal death in a wide variety of neurological conditions [4]. However, attempts to develop anti-excitotoxic agents as neuroprotectants, especially in ischemic stroke, have been unsuccessful, dampening the initial enthusiasm for this unifying mechanism [5]. Instead, recent evidence indicates that multiple mechanisms, including glutamate toxicity, oxidative stress and apoptosis, may act in concert to cause neuronal death in acute brain injury. For instance, glutamate neurotoxicity induces calcium overload [6], which then activates oxidative stress [7]. Reperfusion injury also enhances the production of reactive oxygen species (ROS) [8,9]. In both cases, the resulting increase in oxidative stress causes further glutamate release [10] and excitotoxicity. In addition, calcium-induced apoptosis, inflammation, and autophagy contribute to neuronal death under certain circumstances [3,11].

Endogenous zinc may be another key player in neuronal death following acute brain injury [12]. Like other tissues, the brain contains high levels of intracellular zinc [13]. Because most zinc ions are tightly bound to macromolecules such as nucleic acids and proteins, the level of free zinc in the cytosol is very low, probably in the low nanomolar range [14,15]. The brain, however, has a special pool of zinc in a subset of synaptic vesicles. Glutamatergic synaptic boutons in the forebrain, especially those in the cortical association systems, contain a large amount of free or loosely bound zinc [14]. Although the function of synaptic zinc is still under intensive investigation, a growing body of evidence suggests that similar to glutamate, zinc is released with neuronal activity or membrane depolarization [16-18]. As is the case for glutamate, if a sufficiently high amount of zinc is released during acute brain injury, it can induce neuronal death by entering neurons via calcium-permeable routes [19]. Alternatively, oxidative stress may induce intracellular zinc release from organelles and zinc-binding proteins such as metallothioneins [20,21], again contributing to cell death. Supporting the role of zinc in acute brain injury, several studies have shown that 1) zinc accumulates in degenerating neuronal cells, and 2) blockade of zinc accumulation with chelators substantially reduces neuronal death in diverse models of acute brain injury [22].

Angiotensin II has long been known as a potent vasoconstrictor [23,24]. It is produced from its precursor angiotensinogen by activated angiotensin converting enzyme (ACE) [25]. In addition to its vasoconstrictive effect, angiotensin II induces aldosterone release, sodium and water retention, and increased fluid intake, all of which contribute to blood pressure and fluid homeostasis [26]. Initially, the role of angiotensin II in the brain was not appreciated because angiotensin II does not penetrate the blood–brain barrier (BBB). However, it has since been shown that angiotensin II is produced in the brain, where its production is regulated independently of the classical renin-angiotensin system [27-29]. Hence, it is likely that locally produced angiotensin II may have some parenchymal effects in the brain. For instance, angiotensin II may be involved in the regulation of brain development, neuronal migration, sensory information processing, cognition, emotional responses, and cerebral blood flow [28]. Consistent with this, studies have found that angiotensin II receptors are expressed in neurons as well as in endothelial cells [30,31].

Although the role of angiotensin II in the neuronal death associated with acute brain injury has received little research attention, several lines of evidence suggest that the angiotensin system may be involved in acute neuronal injury. For instance, ischemia-induced neuronal cell loss is accompanied by the loss of angiotensin II receptors. In addition, ACE inhibitors reduce oxidative neuronal death *in vitro* as well as ischemic neuronal death in mice and rats [32]. However, the precise mechanism underlying angiotensin II-related neuroprotection is largely unknown.

Here, using mouse cortical cell cultures, we sought to examine the possibility that the angiotensin system modulates zinc-induced neuronal cell death. We found that angiotensin II potentiates zinc-induced oxidative neuronal death, probably via activation of the angiotensin II type 2 receptor (AT2R) rather than AT1R. In addition, we found that the induction and activation of NADPH oxidase may underlie the oxidative stress-potentiating effects of angiotensin II.

## Results

**Figure 1:**
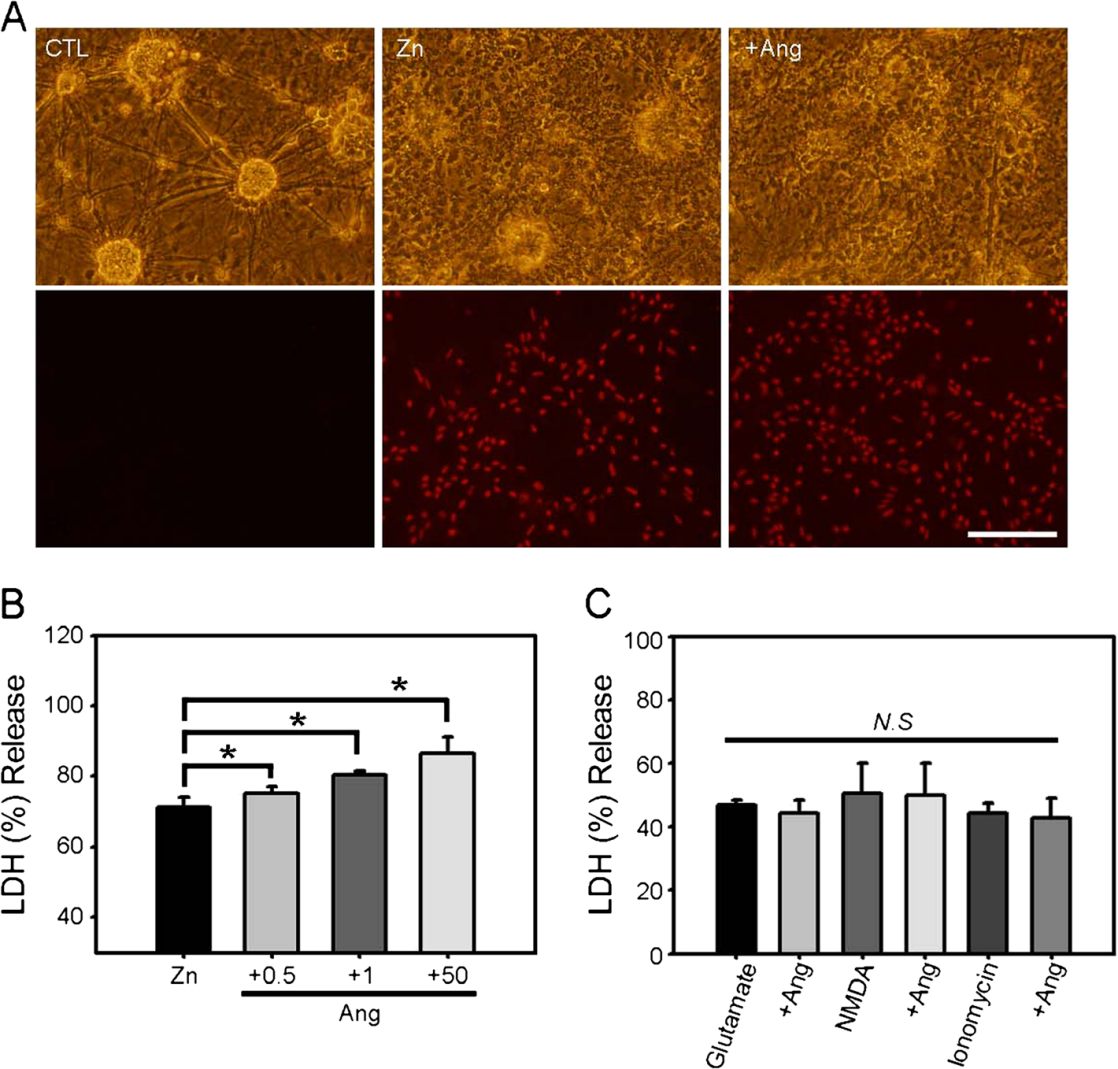
**Angiotensin II potentiates zinc-induced neuronal cell death in cortical cell cultures. A)** Phase-contrast (upper) and matching propidium-iodide fluorescence photomicrographs (lower) of mixed cortical cell cultures after sham wash (CTL), 15 min treatment with 300 μM zinc (Zn), or 15 min treatment with 300 μM zinc plus 1 μM angiotensin II (+Ang). Whereas zinc alone induced a moderate degree of cell death, addition of angiotensin II significantly increased the toxicity, especially to neurons. **B)** LDH release (mean ± SEM, n = 9) in cortical cell cultures after 15 min exposure to 300 μM zinc or zinc plus angiotensin II (0.5–50 μM). Angiotensin II potentiated zinc-triggered cell death in a concentration-dependent manner (*p < 0.05 versus zinc alone; two-tailed t-test). **C)** LDH release in cortical culture after a 24 h exposure to 60 μM glutamate, 20 μM NMDA, or 200 nM ionomycin in the absence or presence of 1 μM angiotensin II. Angiotensin II failed to potentiate calcium-overload excitotoxicity.

Zinc exposure induces concentration-dependent cell death in mixed cortical cultures containing neurons and astrocytes [33]; with 15 min exposure, the 50% lethal dose (LD_50_) of zinc was approximately 300 μM. To analyze the modulating effect of angiotensin II on zinc toxicity in cortical cell cultures, we exposed mixed cortical cultures to 300 μM zinc for 15 min with or without the addition of the indicated concentrations of angiotensin II. As expected, exposure to zinc alone induced about 70% cell death compared to sham wash controls (Figure 1A), whereas exposure to angiotensin II alone at concentrations up to 50 μM for 18 h was not toxic to cultured neurons or astrocytes. In contrast, addition of angiotensin II (0.5–50 μM) significantly enhanced zinc-induced cell death in a concentration-dependent manner (Figure 1B). This potentiating effect was specific for zinc, since addition of 1 μM angiotensin II did not alter the submaximal calcium-overload excitotoxicity induced by 24-h exposure to 60 μM glutamate, 20 μM NMDA, or 200 nM ionomycin (Figure 1C).

**Figure 2:**
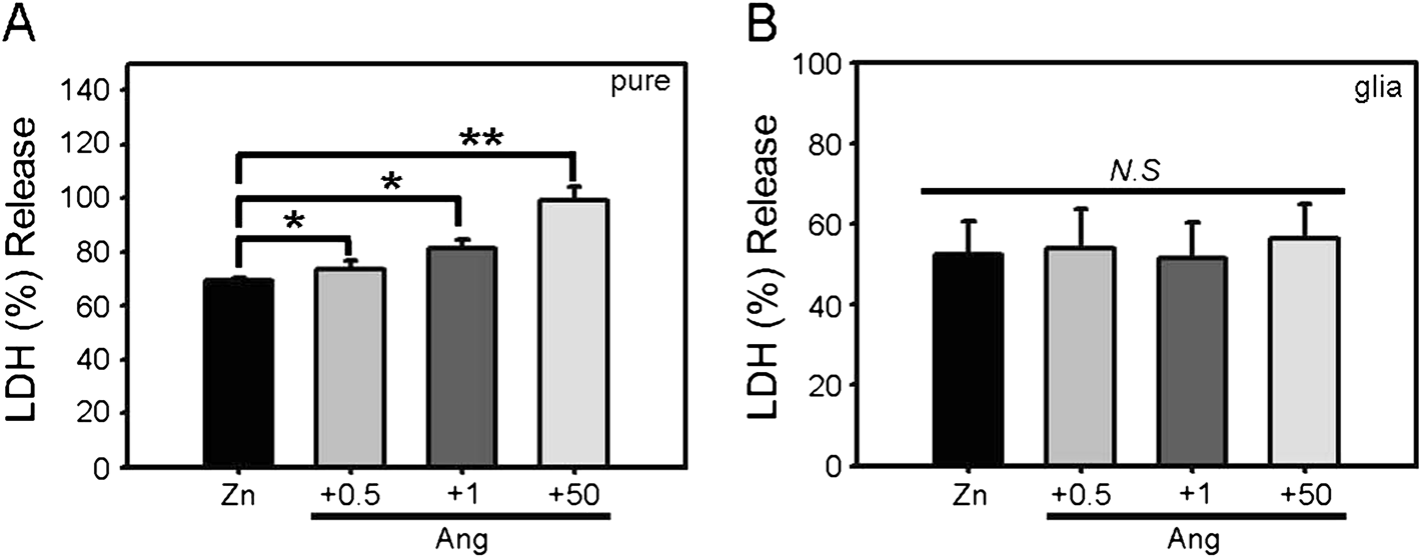
**Potentiation of zinc toxicity by angiotensin II is specific to neurons. A)** Bars denote LDH release (mean ± SEM, n = 7) in near-pure neuronal cultures after 15 min exposure to 300 μM zinc or zinc plus 0.5, 1, or 50 μM angiotensin II (*p < 0.05, **p < 0.01 versus zinc alone; two-tailed t-test). **B)** LDH release (mean ± SEM, n = 7) in astrocytic cultures after 15 min exposure to 300 μM zinc or zinc plus the indicated concentrations of angiotensin II.

Since zinc can injure both neurons and astrocytes, we examined whether the potentiation of zinc-induced cortical cell death by angiotensin II exhibited specificity toward neurons. Separate, near-pure neuronal cultures and astrocytic cultures were prepared, and each culture was exposed to zinc alone (300 μM) or together with angiotensin II (0.5–50 μM). Zinc alone induced about 50% cell death. Addition of angiotensin II significantly potentiated zinc-induced cell death in near-pure neuronal cultures in a concentration-dependent manner (Figure 2A). In contrast, in astrocytic cultures, cell death induced by zinc was not altered by the addition of the same concentrations of angiotensin II (Figure 2B). Hence, the effect of angiotensin II on zinc toxicity appears to be selective for cultured neurons.

**Figure 3:**
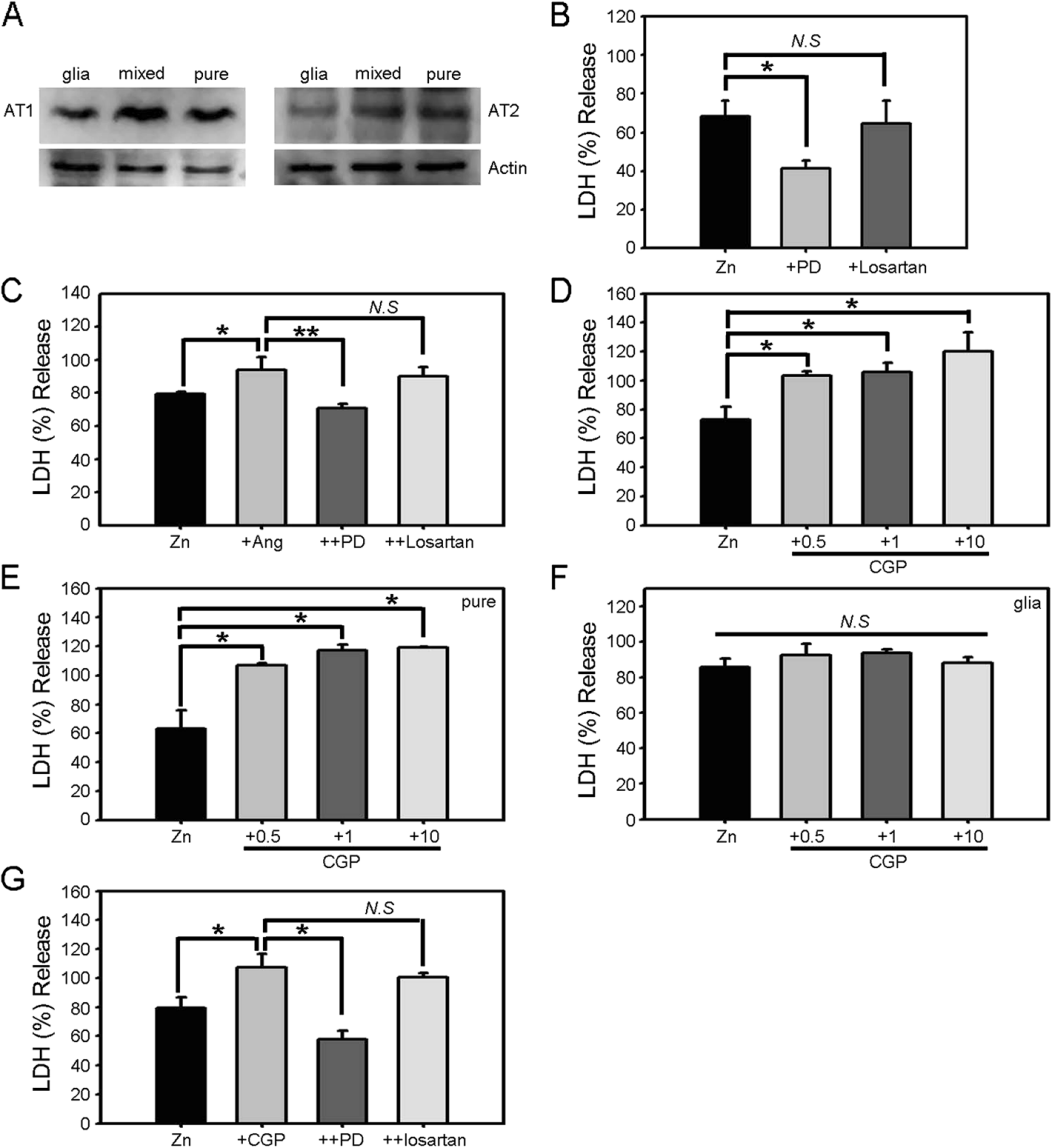
**AT2R mediates zinc toxicity as well as the toxicity-potentiating effect of angiotensin II. A)** Western blots for AT1R and AT2R in astrocytic, mixed, and near-pure neuronal cultures showing that both neurons and astrocytes express substantial levels of both receptors. **B)** LDH release (mean ± SEM, n = 6) in cortical cell cultures after 15 min exposure to 300 μM zinc alone or with addition of the selective AT1R inhibitor losartan (500 nM) or selective AT2R inhibitor PD123319 (1 μM) (*p < 0.05 versus zinc alone; two-tailed t-test). **C)** LDH release (mean ± SEM, n = 5) in cortical cell cultures after 15 min exposure to 300 μM zinc alone, zinc plus 1 μM angiotensin II, zinc plus angiotensin II and losartan, or zinc plus angiotensin II and PD123319 (*p < 0.05 for differences between indicated values; two-tailed t-test). **D)** LDH release (mean ± SEM, n = 4) in cortical cell cultures after 15 min exposure to 300 μM zinc or zinc plus CGP42112 (0.5 μM – 10 μM). CGP42112 potentiated zinc-triggered cell death in a concentration-dependent manner (*p < 0.05 versus zinc alone; two-tailed t-test). **E)** LDH release (mean ± SEM, n = 9) in near-pure neuronal cultures after 15 min exposure to 300 μM zinc or zinc plus 1 μM CGP42112 (*p < 0.05 versus zinc alone; two-tailed t-test). **F)** LDH release (mean ± SEM, n = 6) in astrocytic cultures after 15 min exposure to 300 μM zinc or zinc plus the indicated concentrations of CGP42112. **G)** LDH release (mean ± SEM, n = 10) in cortical cell cultures after 15 min exposure to 300 μM zinc alone, zinc plus 1 μM CGP42112, zinc plus CGP42112 and losartan, or zinc plus CGP42112 and PD123319 (*p < 0.05 for differences between indicated values; two-tailed t-test).

Angiotensin II acts on two types of receptors: type 1 (AT1R) and type 2 (AT2R). To determine whether these receptors are expressed in cultured cortical cells, we performed Western blotting for both receptors in pure neuronal, astrocytic, and mixed cortical cell cultures (Figure 3A). In all cases, both AT1R and AT2R were detected at mRNA and protein levels. We then used losartan and PD123319, selective inhibitors of AT1R and AT2R, respectively, to determine which of the two receptors mediated the potentiating effect of angiotensin II. We first tested the effects of these inhibitors on the neurotoxicity induced by zinc alone. Whereas losartan showed no effect on neuronal death induced by 15 min exposure to 300 μM zinc, PD123319 substantially reduced the cell death (Figure 3B), indicating that activation of AT2Rs contributes to zinc toxicity toward cultured cortical cells. We next examined whether the potentiating effect of angiotensin II on zinc neurotoxicity was mediated by AT1R or AT2R. Again, PD123319, but not losartan, blocked the toxicity-potentiating effect of angiotensin II (Figure 3C), indicating a specific role for AT2Rs.

To further test the distinct role of AT2R in zinc neurotoxicity, we used CGP42112, a specific agonist of AT2R. Co-treatment of CGP42112 increased the neurotoxicity of zinc in a concentration-dependent manner in cortical cell culture (Figure 3D). Similar to angiotensin II, the potentiating effect of CGP42112 was evident in near-pure neuronal culture (Figure 3E) and not in astrocytic culture (Figure 3F). Lastly, we also observed that PD123319 blocked the potentiating effect of CGP42112, while losartan showed no such effect (Figure 3G).

**Figure 4:**
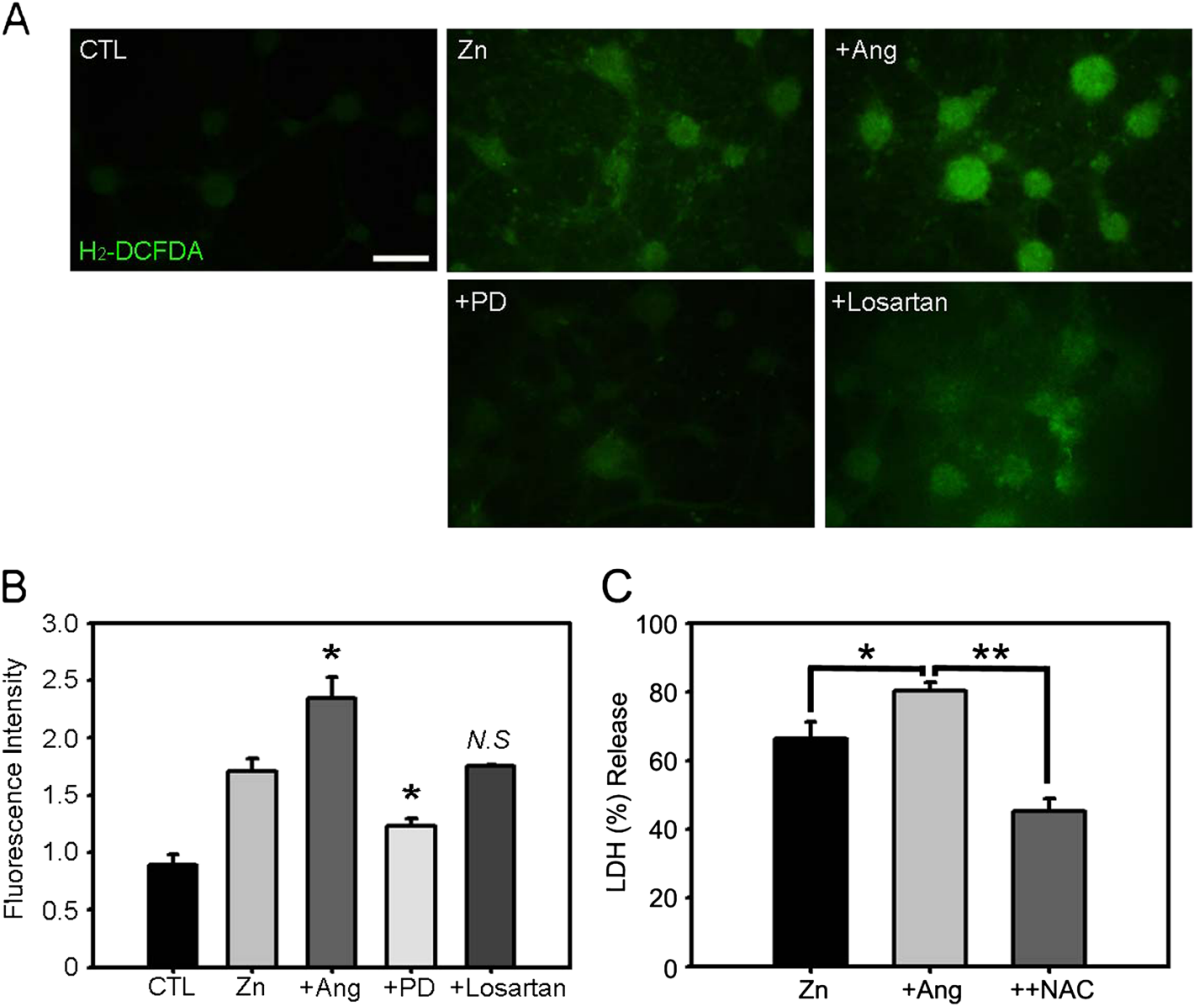
**AT2R modulates the level of zinc-triggered oxidative stress in cortical cells. A)** Cortical cells were loaded with H_2_-DCFDA (DCF) to detect ROS as an indicator of oxidative stress, and subsequently treated with zinc (Zn), zinc plus angiotensin II (+Ang), zinc plus PD123319 (+PD), or zinc plus losartan (+Losartan). Compared to sham-washed cultures, zinc treatment increased the level of DCF fluorescence in neurons, an effect that was further augmented by addition of angiotensin II. PD123319 markedly attenuated the zinc-induced increase in DCF fluorescence in neurons, whereas losartan had no effect. **B)** Bars denote relative DCF fluorescence in cortical cell cultures following 15 min exposure to zinc, zinc plus angiotensin II, zinc plus PD123319, or zinc plus losartan (*p < 0.05 versus zinc alone; two-tailed t-test). **C)** LDH release (mean ± SEM, n = 4) in cortical cell cultures after 15 min exposure to 300 μM zinc, zinc plus angiotensin II (1 μM), and zinc plus angiotensin II and NAC (1 mM). Addition of NAC blocked the potentiating effect of angiotensin II in zinc-induced neuronal cell death (*p < 0.05, **p < 0.01 for differences between indicated values; two-tailed t-test).

Although diverse intracellular events contribute to zinc-triggered cell death [34], oxidative stress is considered a major mechanism [35]. Hence, it is plausible that angiotensin II and AT2Rs specifically participate in oxidative stress mechanisms in the context of zinc neurotoxicity. To examine this possibility, we loaded cortical neurons with H_2_-DCFDA, a fluorescent indicator for superoxides, and examined ROS levels following different treatment regimens. Exposure to 300 μM zinc for 15 min substantially increased the levels of ROS in cultured cortical neurons, an effect that was markedly potentiated by the addition of 1 μM angiotensin II (Figure 4A). Co-incubation with the AT2R inhibitor PD123319 (1 μM) blocked the zinc-induced increases in ROS levels, whereas the AT1R inhibitor losartan was not effective. Quantitative measurements of H_2_-DCFDA fluorescence further confirmed that the inhibition of AT2R specifically reduced ROS levels in zinc-treated cells (Figure 4B). We also used N-acetyl-L-cysteine (NAC) to suppress oxidative stress in zinc-induced neuronal cell death, and found that suppression of oxidative stress fully negated the potentiating effect of angiotensin II (Figure 4C).

**Figure 5:**
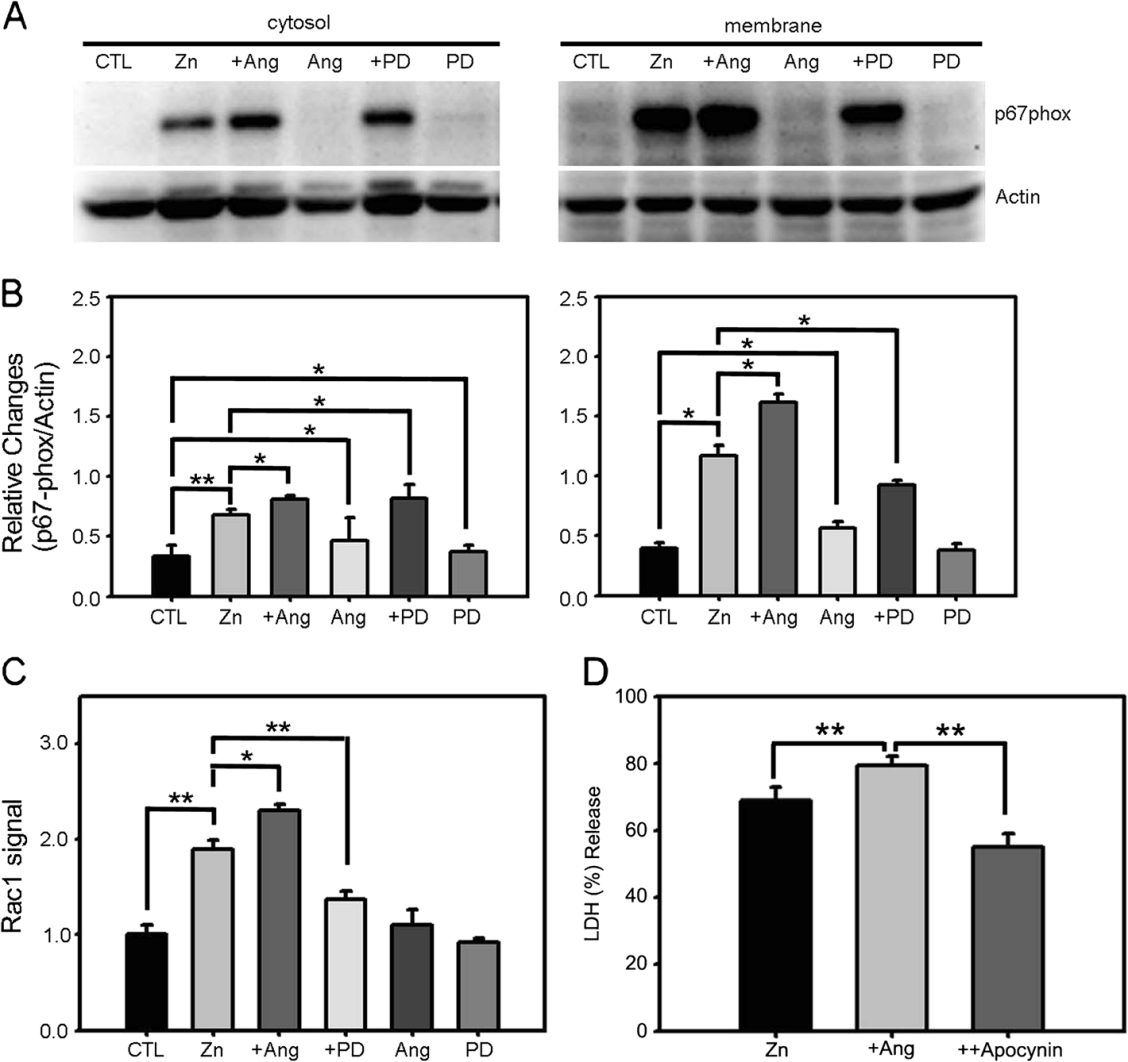
**AT2R modulates zinc-triggered NADPH oxidase activation in cortical cells. A)** Western blotting for the cytosolic subunit of the NADPH oxidase p67^phox^ in cytosolic and membrane fractions of cortical cell cultures, sham-washed (CTL) or after 15 min exposure to 300 μM zinc, zinc plus 1 μM angiotensin II, angiotensin II alone, zinc plus 1 μM PD123319, or PD123319 alone. **B)** Bars denote the relative density of p67^phox^ bands normalized to corresponding actin bands (mean ± SEM, n = 11) in the above Western blot experiments. Whereas angiotensin II or PD123319 alone had no effect on the level or distribution of p67^phox^, zinc exposure substantially increased the levels of p67^phox^ in both cytosolic and membrane fractions, an effect that was significantly potentiated by addition of angiotensin II. Notably, p67^phox^ increased to a greater degree in the membrane fraction, indicating that zinc exposure not only induced, but also activated, NADPH oxidase. Addition of PD123319 inhibited increases in p67^phox^ in the membrane fraction induced by zinc plus angiotensin II (*p < 0.05, **p < 0.01 for differences between indicated values; two-tailed t-test). **C)** Bars denote the level of Rac activation as assessed by G-LISA assay. Consistent with the pattern of NADPH oxidase subunit translocation, zinc treatment increased Rac activity in cortical cells, an effect that was increased by angiotensin II and decreased by PD123319. Again, neither angiotensin II nor PD123319 alone had any effect on the level of Rac activation (*p < 0.05, **p < 0.01 for differences between indicated values; two-tailed t-test). **D)** LDH release (mean ± SEM, n = 4) in cortical cell cultures after 15 min exposure to 300 μM zinc, zinc plus angiotensin II (1 μM), and zinc plus angiotensin II and apocynin (500 μM) (**p < 0.01 for differences between indicated values; two-tailed t-test).

Several studies have demonstrated that NADPH oxidase is a key mediator of ROS generation in zinc neurotoxicity [36]. This enzyme is expressed in neurons and astrocytes in cortical cultures. Furthermore, the levels of NADPH oxidase increase following zinc exposure. Hence, we considered the possibility that NADPH oxidase mediates the effect of angiotensin II, first examining whether angiotensin II alters NADPH oxidase activity in neurons treated with zinc. As we previously reported, 15 min exposure of cortical cells to 300 μM zinc increased the levels of the NADPH subunit p67^phox^ in both cytosolic and membrane fractions, indicating that the total level of p67^phox^ was upregulated by zinc [36]. Notably, p67^phox^ levels increased to a greater degree in the membrane fraction than in the cytosolic fraction, consistent with the membrane translocation of p67^phox^, a characteristic sign of NADPH oxidase activation. Addition of angiotensin II significantly increased the levels of p67^phox^ in both cytosolic and membrane fractions, whereas addition of PD123319 blocked this effect (Figure 5A, B). Angiotensin II alone and PD123319 alone induced no changes in the level or distribution of p67^phox^. Hence, angiotensin II potentiated zinc-triggered NADPH oxidase induction as well as activation, likely through AT2R activation. Further support for this possibility is provided by the results of Rac activation assays, which revealed similar changes. Specifically, whereas zinc induced Rac activation, this activation was potentiated by the addition of angiotensin II and blocked by the addition of PD123319 (Figure 5C). We then tested the causal relationship between the potentiating effect of angiotensin II and NADPH activation by using apocynin, a widely used inhibitor of NADPH oxidase. Indeed, addition of 500 μM apocynin suppressed the potentiating effect of angiotensin II in zinc-induced neuronal cell death (Figure 5D). Collectively, these results indicate that increased activation of NADPH oxidase is among the mechanisms that contribute to the angiotensin II potentiation of zinc-triggered oxidative stress in cortical cultures.

## Discussion

The central finding of the present study is that angiotensin II may modulate the oxidative injury triggered by intracellular zinc dyshomeostasis in cultured cortical neurons. Since the present cortical cell cultures contain no endothelial cells or oligodendrocytes/microglial cells [4,37], this angiotensin II effect is likely mediated by receptors on neurons and/or astrocytes. Our pharmacological data support the possibility that AT2R on neurons is predominantly responsible for this effect.

Mouse cortical cell cultures have been used for more than three decades to study the mechanisms of neuronal death in the central nervous system [4,38]. This primary culture contains mainly of neurons and supporting astrocytes. Compared with pure neuronal or astroglial cultures, this mixed culture may more closely mimic the intact brain, as neuron-astrocytic interactions remain intact. Notably, the results presented here indicate that both neurons and astrocytes express both AT1Rs and AT2Rs. In theory, co-cultured astrocytes could influence neuronal cell fate in this mixed culture. However, the fact that a similar effect was obtained in near-pure neuronal cultures favors the possibility that the effects of angiotensin II are mediated by neuronal angiotensin II receptors.

A signaling role for angiotensin II in the brain was first described decades ago. Previous studies have shown that angiotensin II may be involved in hypothalamic signaling processes, such as hormone regulation and sympathetic control [28]. However, angiotensin II receptors are not confined to the hypothalamus but are instead widely expressed throughout the brain [39], suggesting that angiotensin II may have broader roles in the brain. A growing body of recent evidence suggests that the angiotensin system may directly modulate neuronal cell death associated with acute brain injuries such as ischemia [40]. For instance, ACE inhibitors have been shown to attenuate brain damage in a blood pressure-independent manner [32]. In addition, angiotensin II receptor antagonists have been reported to reduce neuronal cell death in acute brain injury [41], and to reduce apoptosis, inflammation, and oxidative stress in a rat hemorrhagic stroke model [42]. Taken together, these results suggest that the angiotensin system may have direct brain parenchymal effects. In addition, whereas BBB-impermeant ACE inhibitors have little effect on Alzheimer’s disease symptoms, BBB-permeant inhibitors such as perindopril significantly delay the symptomatic progression in Alzheimer’s disease patients [43]. Since the blood pressure-lowering effects were identical in the two groups, this result strongly supports an additional role of the angiotensin system in the brain parenchyma.

Consistent with a role for angiotensin II in the brain parenchyma, the present results indicate that angiotensin II significantly potentiated zinc-triggered neuronal death in cortical cell cultures. We examined zinc toxicity in the present study because a growing body of evidence supports the possibility that zinc dyshomeostasis plays a key role in neuronal death following acute brain injury. First, zinc is present in large quantities in glutamatergic synaptic boutons and is released with neuronal excitation or acute insults such as ischemia [44]. Second, exposure of cultured neurons to millimolar zinc causes neuronal death, mainly via oxidative stress and partially through caspase-mediated apoptosis [35]. Third, following acute brain injuries such as ischemia, seizure or trauma, degenerating neurons exhibit novel zinc accumulation in their cell bodies [45]. Finally, inhibition of zinc accumulation with zinc chelators markedly reduces neuronal cell death in the above conditions [46]. Hence, neuronal death induced by zinc seems a reasonable *in vitro* model for the neuronal death associated with acute brain injury.

Further supporting the role of the angiotensin system in zinc-triggered neuronal death, PD123319, a selective inhibitor of the AT2R, significantly attenuated zinc toxicity in cortical cell culture. In contrast, losartan, a selective inhibitor of AT1Rs, showed no protective effect. Since little information about the angiotensin receptor signaling cascade in brain cells is available, it is unclear how AT2R selectively affects zinc neurotoxicity. In any event, PD123319 blocked the increase in ROS levels in zinc-treated cortical neurons, indicating that AT2R modulates oxidative stress in brain cells under conditions of zinc dyshomeostasis. Again, the fact that similar effects were observed in near-pure neuronal cultures, but not in pure astrocyte cultures, supports the possibility that AT2Rs on neurons likely mediate the effects of angiotensin II reported here. This protective effect exerted by AT2Rs appears to be in conflict with studies reporting a protective effect of an AT2R agonist, CGP42112, in the brain [47]; this difference suggests additional complexities regarding the role of AT2R in brain injury, including the possibility that AT2R may play different roles depending on the mode of cell death.

Several studies have demonstrated that zinc neurotoxicity is mainly caused by oxidative stress [35,48]. Although diverse signaling molecules, such as protein kinase C (PKC) and ERK (extracellular signal-regulated kinase) appear to be important in upstream events, the activation and induction of a superoxide-generating NADPH oxidase is among the main effector mechanisms that directly trigger oxidative neuronal death [36]. NADPH oxidase is mainly expressed in phagocytes; however, recent evidence indicates that this enzyme is expressed more broadly in many types of cells. For instance, in the peripheral nervous system, sympathetic ganglion neurons express NADPH oxidase. In the central nervous system, both neurons and astrocytes, in addition to phagocytic microglial cells, express NADPH oxidase [49]. Interestingly, we found that zinc toxicity, but not calcium-overload glutamate toxicity, selectively activated and induced NADPH oxidase in cortical cell cultures [36]. Consistent with this, angiotensin II also selectively potentiated zinc toxicity through activation of NADPH oxidase; again, calcium-overload excitotoxicity was not altered by angiotensin II. Consistently, we also found that NAC and apocynin inhibited the potentiating effect of angiotensin II, thus further supporting the idea that angiotensin II exerts its death-potentiating effect through activation of NADPH oxidase and ROS production. In this context, it is intriguing that angiotensin II augmented the zinc-induced increase in NADPH oxidase subunit in cortical cell cultures by acting through AT2R (reversed by addition of PD123319). Although deconvoluting the entire signaling cascade from AT2R activation to NADPH oxidase activation/induction is beyond the scope of this study, it is likely that PKC activation is involved [50].

## Conclusions

The present study demonstrates a role for the angiotensin system in zinc-triggered neuronal cell death. Since zinc toxicity is likely a key component in neuronal death associated with acute brain injury in diverse animal models, the use of BBB-permeable ACE inhibitors or angiotensin II receptor antagonists may provide neuroprotection in some cases of acute brain injury.

## Methods

### Cortical cell cultures

Mixed cortical cell cultures containing both neurons and astrocytes were prepared from fetal mice at day 15 of gestation. Briefly, dissociated cortical cells were plated onto a previously established astroglial cell monolayer at 5 hemispheres per plate (Nunc, Roskilde, Denmark) in plating medium (Dulbecco’s modified Eagle medium; Gibco BRL, Rockville, MD, USA) supplemented with 20 mM glucose, 38 mM sodium bicarbonate, 2 mM glutamine, 5% fetal bovine serum, and 5% horse serum. Cytosine arabinoside (10 μM) was added 5–6 d after plating to halt the growth of non-neuronal cells.

Astroglial cultures were prepared from neocortices of newborn mice (postnatal day 1–3) and plated at 2 hemispheres per plate in the same plating medium indicated above but supplemented with 7% fetal bovine serum and 7% horse serum. Glial cultures were used for neuronal plating between days *in vitro* (DIV) 14 and 28, when they had formed a confluent monolayer.

### LDH release assay

Overall neuronal cell injury in mixed cortical cultures was quantitatively assessed by measuring lactate dehydrogenase (LDH; EC 1.1.1.27) activity released from damaged cells into the culture medium [51]. Each LDH value was scaled to the mean value in sister positive control cultures treated with 300 μM glutamate for 24 h (defined as 100%) after subtracting the mean background value in sister control untreated cultures that underwent sham wash only (defined as 0%). The positive control (300 μM glutamate) induced near-complete neuronal death without producing glial damage.

### Measurement of intracellular ROS levels by DCF fluorescence microscopy

Levels of intracellular free radicals were measured using the peroxide-sensitive fluorescent probe, 5-(and-6)-carboxy-2′,7′-dichlorohydrofluorescein diacetate (H_2_-DCFDA), as described by the manufacturer (Molecular Probes, Eugene, OR, USA). Briefly, cells were incubated for 10 min in the presence of 5 μM H_2_-DCFDA (DCF), washed with minimal essential medium (MEM), and viewed and photographed under a fluorescence microscope.

### Measurement of Rac activity

Rac activation was measured using the G-LISA Rac activation assay Biochem Kit (Cytoskeleton Inc. CO, USA) as described by the manufacturer. Briefly, cell lysates were incubated in a Rac-GTP affinity plate for 30 min, and active Rac was detected with a Rac-specific antibody.

### Membrane-cytosol fractionation for detection of the NADPH oxidase component p67^phox^

Membrane translocation of the NADPH oxidase component p67^phox^ was detected by Western blot analysis of membrane/cytosol-fractionated cell lysates. Cells were lysed and collected using hypotonic lysis buffer (1 mM NaHCO_3_ and 5 mM MgCl_2_ in RIPA buffer), sonicated briefly, and centrifuged at 600 × g for 10 min at 4°C. The membrane fraction was separated from the cytosol fraction by centrifuging the supernatant at 55,000 rpm for 1.5 h in an Optima TLX ultracentrifuge (Beckman Coulter, CA, USA). After centrifugation, the resulting supernatant (cytosol fraction) was transferred to a separate tube. The pellet (membrane fraction) was incubated on ice in RIPA buffer for 30 min, and then sonicated and centrifuged at 12,000 rpm for 5 min in at 4°C. The supernatant of this final centrifugation step was collected as the membrane fraction. p67^phox^ was detected and quantified in both cytosol and membrane fractions by Western blotting.

## Abbreviations

ACE: Angiotensin converting enzyme; AT1R: Angiotensin II type 1 receptor; AT2R: Angiotensin II type 2 receptor; BBB: Blood–brain barrier; DCF: Dichlorohydrofluorescein; ERK: Extracellular signal-regulated kinase; LDH: Lactate dehydrogenase; PKC: Protein kinase C; ROS: Reactive oxygen species.

## Competing interests

The authors declare that they have no competing interests.

## Authors’ contributions

MHP, HNK, JSL and JSA designed and performed the experiments, analyzed the data and wrote the manuscript. JYK supervised the experiments and wrote the manuscript. All authors read and approved the final manuscript.
